# Primary Vitamin D Target Genes of Human Monocytes

**DOI:** 10.3389/fphys.2019.00194

**Published:** 2019-03-05

**Authors:** Veijo Nurminen, Sabine Seuter, Carsten Carlberg

**Affiliations:** ^1^School of Medicine, Institute of Biomedicine, University of Eastern Finland, Kuopio, Finland; ^2^Institute for Cardiovascular Physiology, Medical Faculty, Goethe University Frankfurt, Frankfurt, Germany

**Keywords:** vitamin D, VDR, epigenome, transcriptome, gene regulation, vitamin D target genes, monocytes

## Abstract

The molecular basis of vitamin D signaling implies that the metabolite 1α,25-dihydroxyvitamin D_3_ (1,25(OH)_2_D_3_) of the secosteroid vitamin D_3_ activates the transcription factor vitamin D receptor (VDR), which in turn modulates the expression of hundreds of primary vitamin D target genes. Since the evolutionary role of nuclear receptors, such as VDR, was the regulation of cellular metabolism, the control of calcium metabolism became the primary function of vitamin D and its receptor. Moreover, the nearly ubiquitous expression of VDR enabled vitamin D to acquire additional physiological functions, such as the support of the innate immune system in its defense against microbes. Monocytes and their differentiated phenotypes, macrophages and dendritic cells, are key cell types of the innate immune system. Vitamin D signaling was most comprehensively investigated in THP-1 cells, which are an established model of human monocytes. This includes the 1,25(OH)_2_D_3_-modulated cistromes of VDR, the pioneer transcription factors PU.1 and CEBPA and the chromatin modifier CTCF as well as of the histone markers of promoter and enhancer regions, H3K4me3 and H3K27ac, respectively. These epigenome-wide datasets led to the development of our chromatin model of vitamin D signaling. This review discusses the mechanistic basis of 189 primary vitamin D target genes identified by transcriptome-wide analysis of 1,25(OH)_2_D_3_-stimulated THP-1 cells and relates the epigenomic basis of four different regulatory scenarios to the physiological functions of the respective genes.

## Introduction

In all animal species, in which the cholesterol precursor 7-dehydrocholesterol is exposed to UV-B, the secosteroid vitamin D_3_ is formed in a non-enzymatic reaction (Hart et al., 2011). At a sunny day with an UV index of 3 or higher humans can produce vitamin D_3_ in their unprotected skin (Holick, 2011). However, today’s lifestyle with predominant indoor activity and textile coverage outdoors as well as seasonal variations in sun intensity at latitudes higher than 30°N or lower than 30°S often prevents endogenous vitamin D_3_ synthesis and makes the molecule for a large proportion of the human population an essential micronutrient, i.e., a true vitamin, that needs to be taken up by diet or supplementation with pills (Bendik et al., 2014; Carlberg, 2016). Interestingly, more than 100 years ago a sunshine cure leading to endogenous production of vitamin D_3_ was used as an efficient therapy of the children’s bone malformation disease rickets as well as of the infectious disease tuberculosis (Holick, 1981; Grad, 2004).

Vitamin D_3_ itself is biologically inert, but hydroxylation at carbon 25 of its side chain into 25-hydroxyvitamin D_3_ and further hydroxylation at carbon 1 within its A-ring results in the active metabolite 1,25(OH)_2_D_3_. As a lipophilic molecule 1,25(OH)_2_D_3_ easily passes through biological membranes and binds with high-affinity (k_D_ 0.1 nM) to the transcription factor VDR, which is primarily located in the nucleus (Haussler et al., 2013). This explains why vitamin D_3_ has via its metabolite 1,25(OH)_2_D_3_ major effects on the transcriptome and the resulting proteome of VDR-expressing cell types. Proximal tubule cells of the kidneys are the main production site of the circulating endocrine hormone 1,25(OH)_2_D_3_, but for para- and autocrine use the molecule is also formed in monocytes, macrophages and dendritic cells of the innate immune system, osteoblasts within bones and keratinocytes of the skin (Hewison, 2012).

When 250–500 million years ago some animal species left the ocean and had to develop a stable calcium-based skeleton, 1,25(OH)_2_D_3_ developed to a nuclear hormone that, via its receptor, took over the role as main regulator of calcium homeostasis (Bouillon and Suda, 2014). Within the same evolutionary time span, the immune system of vertebrates further evolved and the vitamin D endocrine system acquired a role in its regulation, in order to mediate a more efficient protection against infectious diseases, such as tuberculosis. Tuberculosis is caused by the intra-cellular bacterium *Mycobacterium tuberculosis*, the proliferation of which within macrophages is inhibited by vitamin D (Rook et al., 1986). Moreover, after vitamin D treatment monocytes and macrophages recognize bacterial pathogens more efficiently via pattern-recognition receptors, such as toll-like receptors (Liu et al., 2006). A key player in the anti-microbial effects of 1,25(OH)_2_D_3_ is the protein cathelicidin, which is encoded by the primary vitamin D target gene *CAMP* and rapidly destroys the lipoprotein membranes of microbes (Gombart et al., 2005). Transcriptome-wide analysis indicated that in human monocytes a few hundred additional genes respond to vitamin D (Seuter et al., 2016). This review will present the epigenome- and transcriptome-wide response of the monocytic cell line THP-1 to vitamin D as a paradigm for distinguishing four regulatory scenarios of vitamin D target genes.

## The Transcription Factor VDR

Larger amounts of VDR protein are found in intestine, kidneys, skin, parathyroid gland and pituitary gland, but also most of the other 400 tissues and cell types of the human body, including those of the innate and adaptive immune system, show some VDR expression^1^. Since transcription factors, such as VDR, do not need high expression levels for their effective function, it can be assumed that most human tissues are sensitive to vitamin D.

Like other members of the nuclear receptor superfamily, VDR carries a structurally conserved ligand-binding domain, the inner surface of which forms a ligand-binding pocket that snugly encloses the molecule 1,25(OH)_2_D_3_ (Molnár et al., 2006). In turn, VDR interacts via the outer surface of the ligand-binding domain with other nuclear proteins, such as histone acetyltransferases (Herdick and Carlberg, 2000), co-repressors contacting histone deacetylases (Polly et al., 2000), lysine demethylases (Pereira et al., 2011) and chromatin remodelers (Wei et al., 2018). These proteins either form large protein complexes with VDR or their genes are primary or secondary targets of vitamin D.

**FIGURE 1:**
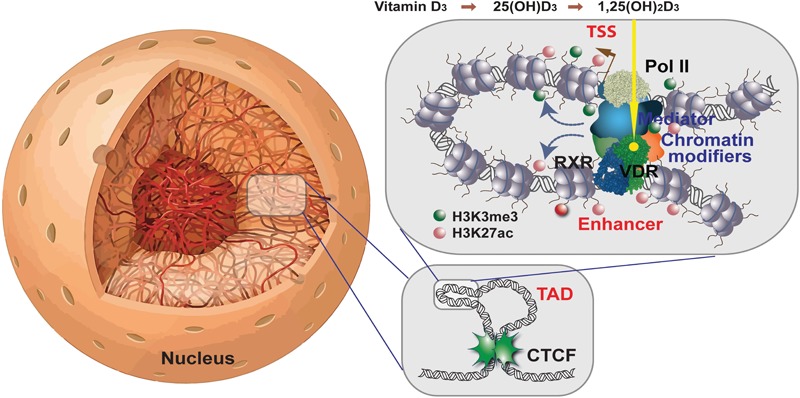
Vitamin D signaling in the context of chromatin. Chromatin within the nucleus forms a 3D architecture **(left)**. Two CTCF proteins bound at adjacent chromatin boundaries form a complex defining a TAD **(right bottom)**. Enhancers and TSS regions that are located within the same TAD can get into physical contact within DNA looping **(right top)**. 1,25(OH)_2_D_3_-activated VDR forms a heterodimeric complex with RXR on enhancer regions carrying appropriate binding sites. In this way, chromatin modifiers are activated that change histone marks (shown here are H3K4me3 modifications marking active TSS regions and H3K27ac indicating active chromatin) and the mediator complex forms a bridge to the basal transcriptional machinery with RNA polymerase II as its core. This finally leads to mRNA transcription of respective vitamin D target genes.

The special feature of transcription factors, such as VDR, is their ability to bind in a sequence-specific fashion to genomic DNA. VDR preferentially binds as a heterodimeric complex with its partner nuclear receptor retinoid X receptor (RXR) to a direct repeat of the hexameric motif A/GGG/TTC/GA spaced by three nucleotides, which is referred to as a DR3-type response element (Carlberg et al., 1993; Figure 1). The genome-wide binding pattern of VDR, its so-called cistrome, was determined by the method chromatin immunoprecipitation combined with massive parallel sequencing (ChIP-seq). In human the VDR cistrome had been figured out in lymphocytes (Ramagopalan et al., 2010), colorectal cancer cells (Meyer et al., 2012), hepatic stellate cells (Ding et al., 2013) and macrophage-like cells (Tuoresmäki et al., 2014), but the most comprehensive analysis was done for the monocytic cell line THP-1 (Heikkinen et al., 2011; Neme et al., 2017). After stimulation with VDR ligand in all these *in vitro* cell culture models some 5,000–20,000 genomic binding sites were observed, which is a 2- to 10-fold increase compared to the respective basal condition. Interestingly, the VDR cistrome in THP-1 cells contains a few hundred persistent loci that stay always occupied (Neme et al., 2017). These primary contacts of the human genome with 1,25(OH)_2_D_3_ are considered as “hotspots” of vitamin D signaling that coordinate the functional consequences of a stimulation with vitamin D over time. In addition, there are transient VDR binding loci that modulate the response of the epigenome to vitamin D and support persistent VDR sites. Thus, the genome-wide, ligand-induced binding of VDR to its preferred loci represents the most prominent epigenome-wide effect of vitamin D.

## The Chromatin Model of Vitamin D Signaling

Chromatin is the complex of genomic DNA with nucleosomes that stabilizes the epigenetic landscape of a differentiated cell (Beisel and Paro, 2011). By default chromatin largely restricts the access of transcription factors to promoter and enhancer regions, so that per cell type only some 100–200,000 genomic regions are accessible (The Encode-Project-Consortium et al., 2012). The epigenome is represented by (i) more than 100 post-translational modifications, such as acetylations and methylations, of the four nucleosome-forming histone proteins (Carlberg and Molnár, 2016a), (ii) DNA methylation, preferentially within so-called “CpG islands” (Dawson and Kouzarides, 2012) and iii) higher order chromatin structures, such as the three-dimensional organization of the genome into topologically associated domains (TADs) (Carlberg and Molnár, 2016b). Importantly, the epigenome is dynamic, i.e., it can be modulated by environmental signals that influence chromatin modifying enzymes, such as DNA methyltransferases, histone acetyl- and methyltransferases as well as histone deacetylases and demethylases, that write, read or erase marks on genomic DNA and histone proteins.

For the transcription of a gene it is essential that the genomic regions of both its transcription start site (TSS) and the binding sites of the transcription factors controlling the activity of RNA polymerase II, referred to as enhancers, are located within accessible chromatin (Carlberg and Campbell, 2013). Open chromatin can be measured by the method formaldehyde-assisted isolation of regulatory elements sequencing (FAIRE-seq) and some 9,000 sites of accessible chromatin were found to be sensitive to vitamin D stimulation (Seuter et al., 2016). A prominent marker of active chromatin at enhancer regions is the histone modification H3K27ac, while H3K4me3 labels TSS regions. Interestingly, both types of histone markers are sensitive to vitamin D (Nurminen et al., 2018).

In human, most epigenome-wide data on 1,25(OH)_2_D_3_ and its receptor VDR have been collected in cells of the innate and adaptive immune system (Carlberg, 2014). Accordingly, the chromatin model of vitamin D signaling, describing the sequential process of vitamin D target gene activation, has been developed based on epigenome- and transcriptome-wide data obtained primarily in THP-1 cells (Carlberg, 2017). As outlined above, the first event in vitamin D signaling is the genome-wide binding of ligand-activated VDR molecules to enhancer regions that carry suitable binding motifs and are located within accessible chromatin. With the help of pioneer factors, such as PU.1 (Seuter et al., 2017a), CEBPA (Nurminen et al., 2019) and GABPA (Seuter et al., 2018) VDR increases the accessibility of chromatin at and around these enhancer regions. This epigenomic process also involves local changes in the intensity of H3K27ac histone markers marking active chromatin at enhancer and TSS regions (Nurminen et al., 2018). Via DNA looping VDR-bound, activate enhancer regions contact TSS regions within the same TAD and trigger the transcription of the respective vitamin D target genes (Figure 1).

TADs range in size from 30 kb to 3 Mb and subdivide the human genome into at least 2,000 functionally independent domains (Dixon et al., 2012). Enhancer-TSS contacts are very likely within the same TAD, while interaction with genomic regions outside of a TAD are inhibited by insulating TAD borders that are marked by the transcription factor CTCF (Ali et al., 2016; Figure 1). Thus, VDR-bound enhancers can only modulate the transcription of those genes, which are located within the same TAD. Accordingly, the linear distance between a VDR site within an enhancer and its target TSS is limited by the size of the respective TAD but it can be many hundred kb (see Supplementary Table S1 for examples).

Interestingly, in THP-1 cells 1,25(OH)_2_D_3_ stimulation significantly (*p* < 0.05) affects the binding strength of CTCF to TAD anchors making some 600 TADs sensitive of vitamin D (Neme et al., 2016b). Looping of activated DNA-bound VDR to a TSS leads at these promoter regions to an increase in chromatin accessibility as well as of H3K27ac and H3K4me3 marks (Seuter et al., 2016; Nurminen et al., 2019). All these vitamin D-triggered changes in the local chromatin structure at enhancer and promoter regions finally lead to the activation of RNA polymerase II assembled on the respective TSSs and the start of mRNA synthesis. Thus, vitamin D signaling involves a number of epigenome-wide events before there are responses on the level of the transcriptome.

The chromatin model of vitamin D signaling explains the activation of primary vitamin D target genes, which are the focus of this review. However, there is also a large number of secondary vitamin D target genes, the activation of which do not directly require VDR as a central protein. The activity of transcription factors other than VDR and/or chromatin modifying proteins, which are encoded by primary targets of vitamin D, mediate the activation of these secondary vitamin D target genes. Examples are the transcription factors BCL6, NFE2, POU4F2, and ELF4, which are in THP-1 cells primary vitamin D target genes (Nurminen et al., 2015).

## Vitamin D Target Genes in THP-1 Cells

Since more than two decades many research groups have used the human acute monocytic leukemia cell line THP-1 (Tsuchiya et al., 1980) as a model system for investigating the effects of vitamin D-triggered physiological processes in the context of innate immunity. A PubMed search (September 19, 2018) with the keywords “Vitamin D AND THP-1” revealed 161 publications, which were manually inspected whether they report in undifferentiated THP-1 cells statistically significant effects of 1,25(OH)_2_D_3_ on the mRNA expression of individual genes, as measured by qPCR, or of gene sets, as monitored by microarrays or RNA sequencing (RNA-seq). The attribute “undifferentiated THP-1 cells” was fulfilled by 53 articles, 19 of which were not considered, since they used less quantitative assays, such as Northern blotting, or focused on changes in protein expression by flow cytometry or Western blotting. The remaining 34 publications reported effects of 1,25(OH)_2_D_3_ on enhancer/TSS regions and/or mRNA expression of in total 107 different genes (Table 1). In reference to a re-analyzed RNA-seq time course (Seuter et al., 2016; Neme et al., 2017; Nurminen et al., 2019), of these genes were classified as primary vitamin D targets, i.e., their expression changed significantly (*p* < 0.05) within 4 h after onset of stimulation with 1,25(OH)_2_D_3_, while 28 genes were secondary vitamin D targets. However, the transcriptome-wide assay failed to confirm 15 genes, i.e., 14% of all, as vitamin D target genes. This reflects a well-known discrepancy, which is based mainly on different threshold settings and more strict statistical approaches of the transcriptome-wide method.

**Table 1:** Vitamin D target genes.

Gene	Primary?	Type(s) of assays	Citation
*CAMP*	Yes	qPCR	Liu et al., 2007
*CYP24A1*	–	qPCR	Liu et al., 2007
*CYP24A1*	–	qPCR	Wu et al., 2007
*CYP27B1*	–	qPCR	Wu et al., 2007
*CD14*	Yes	qPCR	Moeenrezakhanlou et al., 2008
*ITGAM*	Yes	qPCR	Moeenrezakhanlou et al., 2008
*ALOX5*	No	qPCR	Matsunawa et al., 2009
*CAMP*	Yes	qPCR	Matsunawa et al., 2009
*CD14*	Yes	qPCR	Matsunawa et al., 2009
*CYP24A1*	*–*	qPCR	Matsunawa et al., 2009
*FANCE*	Yes	ChIP-seq & FAIRE-seq display, qPCR	Seuter et al., 2013b
*HBEGF*	Yes	ChIP-seq & FAIRE-seq display, qPCR	Seuter et al., 2013b
*NFKBIA*	Yes	ChIP-seq & FAIRE-seq display, qPCR	Seuter et al., 2013b
*PDCD1LG2*	Yes	ChIP-seq & FAIRE-seq display, qPCR	Seuter et al., 2013b
*TMEM37*	Yes	ChIP-seq & FAIRE-seq display, qPCR	Seuter et al., 2013b
*BHLHE40*	*–*	ChIP-seq & FAIRE-seq display, qPCR	Seuter et al., 2013c
*CAMP*	Yes	ChIP-seq & FAIRE-seq display, qPCR	Seuter et al., 2013a
*CD93*	Yes	ChIP-seq & FAIRE-seq display, qPCR	Seuter et al., 2013a
*DUSP10*	Yes	ChIP-seq & FAIRE-seq display, qPCR	Seuter et al., 2013a
*HBEGF*	Yes	ChIP-seq & FAIRE-seq display, qPCR	Seuter et al., 2013a
*NFKBIA*	Yes	ChIP-seq & FAIRE-seq display, qPCR	Seuter et al., 2013a
*THBD*	Yes	ChIP-seq & FAIRE-seq display, qPCR	Seuter et al., 2013a
*IL1B*	No	qPCR, microarray	Verway et al., 2013
*ASAP2*	Yes	ChIP-seq display, qPCR	Seuter et al., 2014b
*YWHAQ*	No	ChIP-seq display, qPCR	Seuter et al., 2014b
*SEPT3*	Yes	ChIP-seq & FAIRE-seq display, qPCR	Seuter et al., 2014a
*SFT2D1*	Yes	ChIP-seq & FAIRE-seq display, qPCR	Seuter et al., 2014a
*SP100*	Yes	ChIP-seq & FAIRE-seq display, qPCR	Seuter et al., 2014a
*ZFP36*	Yes	ChIP-seq & FAIRE-seq display, qPCR	Seuter et al., 2014a
*TNF*	*–*	qPCR	Reeves et al., 2014
*ASAP2*	Yes	ChIP-seq display	Tuoresmäki et al., 2014
*CAMP*	Yes	ChIP-seq display	Tuoresmäki et al., 2014
*DENND6B*	Yes	ChIP-seq display	Tuoresmäki et al., 2014
*NINJ1*	Yes	ChIP-seq display	Tuoresmäki et al., 2014
*PTGER3*	–	ChIP-seq display	Tuoresmäki et al., 2014
*SP100*	Yes	ChIP-seq display	Tuoresmäki et al., 2014
*TBP*	No	ChIP-seq display	Tuoresmäki et al., 2014
*TRAK1*	Yes	ChIP-seq display	Tuoresmäki et al., 2014
*TLR10*	*–*	qPCR	Verma et al., 2014
*CD97*	Yes	ChIP-seq & FAIRE-seq display, qPCR	Wilfinger et al., 2014
*LRRC8A*	Yes	ChIP-seq & FAIRE-seq display, qPCR	Wilfinger et al., 2014
*NRIP1*	Yes	ChIP-seq & FAIRE-seq display, qPCR	Wilfinger et al., 2014
*SLC37A2*	Yes	ChIP-seq & FAIRE-seq display, qPCR	Wilfinger et al., 2014
*TREM1*	Yes	qPCR	Hosoda et al., 2015
*BCL6*	Yes	Microarray, qPCR	Nurminen et al., 2015
*ELF4*	Yes	ChIP-seq & FAIRE-seq display, microarray, qPCR	Nurminen et al., 2015
*FUCA1*	Yes	ChIP-seq & FAIRE-seq display, microarray, qPCR	Nurminen et al., 2015
*ITGAM*	Yes	ChIP-seq & FAIRE-seq display, microarray, qPCR	Nurminen et al., 2015
*LPGAT1*	No	ChIP-seq & FAIRE-seq display, microarray, qPCR	Nurminen et al., 2015
*LPP*	*–*	Microarray, qPCR	Nurminen et al., 2015
*LRRC25*	Yes	ChIP-seq & FAIRE-seq display, microarray, qPCR	Nurminen et al., 2015
*NFE2*	Yes	ChIP-seq & FAIRE-seq display, microarray, qPCR	Nurminen et al., 2015
*POU4F2*	Yes	ChIP-seq & FAIRE-seq display, microarray, qPCR	Nurminen et al., 2015
*RTP4*	*–*	Microarray, qPCR	Nurminen et al., 2015
*SHE*	Yes	ChIP-seq & FAIRE-seq display, microarray, qPCR	Nurminen et al., 2015
*SLC45A3*	No	ChIP-seq & FAIRE-seq display, microarray, qPCR	Nurminen et al., 2015
*TMEM243*	No	ChIP-seq & FAIRE-seq display, microarray, qPCR	Nurminen et al., 2015
*TREM1*	Yes	ChIP-seq & FAIRE-seq display, microarray, qPCR	Nurminen et al., 2015
*CD274*	Yes	ChIP-seq & FAIRE-seq display	Neme et al., 2016b
*CYP26B1*	Yes	ChIP-seq & FAIRE-seq display	Neme et al., 2016b
*EPB41L1*	No	ChIP-seq & FAIRE-seq display	Neme et al., 2016b
*PLGRKT*	No	ChIP-seq & FAIRE-seq display	Neme et al., 2016b
*SIRT4*	Yes	ChIP-seq & FAIRE-seq display	Neme et al., 2016b
*APBB3*	No	ChIP-seq & FAIRE-seq display	Neme et al., 2016a
*CCL2*	Yes	ChIP-seq & FAIRE-seq display	Neme et al., 2016a
*CD14*	Yes	ChIP-seq & FAIRE-seq display	Neme et al., 2016a
*FCER2*	No	ChIP-seq & FAIRE-seq display	Neme et al., 2016a
*HBEGF*	Yes	ChIP-seq & FAIRE-seq display	Neme et al., 2016a
*PFDN1*	Yes	ChIP-seq & FAIRE-seq display	Neme et al., 2016a
*SLC35A4*	Yes	ChIP-seq & FAIRE-seq display	Neme et al., 2016a
*SRA1*	Yes	ChIP-seq & FAIRE-seq display	Neme et al., 2016a
*WDR55*	Yes	ChIP-seq & FAIRE-seq display	Neme et al., 2016a
*ALOX5*	No	qPCR, RNA-seq	Seuter et al., 2016
*ASAP2*	Yes	qPCR, RNA-seq	Seuter et al., 2016
*CAMP*	Yes	qPCR, RNA-seq	Seuter et al., 2016
*CD14*	Yes	qPCR, RNA-seq	Seuter et al., 2016
*ELL*	Yes	qPCR, RNA-seq	Seuter et al., 2016
*FANCE*	Yes	qPCR, RNA-seq	Seuter et al., 2016
*FBP1*	Yes	qPCR, RNA-seq	Seuter et al., 2016
*G0S2*	Yes	qPCR, RNA-seq	Seuter et al., 2016
*HBEGF*	Yes	qPCR, RNA-seq	Seuter et al., 2016
*HTT*	Yes	ChIP-seq & FAIRE-seq display, RNA-seq	Seuter et al., 2016
*MPC1*	No	qPCR, RNA-seq	Seuter et al., 2016
*MYC*	*–*	qPCR, RNA-seq	Seuter et al., 2016
*NOD2*	Yes	ChIP-seq & FAIRE-seq display, RNA-seq	Seuter et al., 2016
*PPARGC1B*	Yes	qPCR, RNA-seq	Seuter et al., 2016
*THBD*	Yes	qPCR, RNA-seq	Seuter et al., 2016
*TMEM37*	Yes	qPCR, RNA-seq	Seuter et al., 2016
*ZFP36*	Yes	qPCR, RNA-seq	Seuter et al., 2016
*CYTH4*	Yes	ChIP-seq & FAIRE-seq display	Carlberg, 2017
*ELFN2*	Yes	ChIP-seq & FAIRE-seq display	Carlberg, 2017
*AKIRIN1*	Yes	ChIP-seq & FAIRE-seq display	Seuter et al., 2017a
*BDH1*	No	ChIP-seq & FAIRE-seq display	Seuter et al., 2017a
*CAMP*	Yes	ChIP-seq & FAIRE-seq display	Seuter et al., 2017a
*CD14*	Yes	ChIP-seq & FAIRE-seq display	Seuter et al., 2017a
*CD226*	No	ChIP-seq & FAIRE-seq display	Seuter et al., 2017a
*CD274*	Yes	ChIP-seq & FAIRE-seq display, qPCR	Seuter et al., 2017a
*CD36*	Yes	qPCR	Seuter et al., 2017a
*CD97*	Yes	ChIP-seq & FAIRE-seq display	Seuter et al., 2017a
*DRAM1*	No	ChIP-seq & FAIRE-seq display	Seuter et al., 2017a
*DUSP10*	Yes	ChIP-seq & FAIRE-seq display	Seuter et al., 2017a
*FANCE*	Yes	qPCR	Seuter et al., 2017a
*FHL1*	No	ChIP-seq & FAIRE-seq display	Seuter et al., 2017a
*MYO7B*	No	ChIP-seq & FAIRE-seq display	Seuter et al., 2017a
*PCTP*	No	ChIP-seq & FAIRE-seq display	Seuter et al., 2017a
*PPARGC1B*	Yes	ChIP-seq & FAIRE-seq display	Seuter et al., 2017a
*RTCB*	*–*	ChIP-seq & FAIRE-seq display	Seuter et al., 2017a
*SLC37A2*	Yes	ChIP-seq & FAIRE-seq display	Seuter et al., 2017a
*SPI1*	*–*	qPCR	Seuter et al., 2017a
*AGPAT1*	Yes	ChIP-seq & FAIRE-seq display	Carlberg, 2018
*CD14*	Yes	ChIP-seq & FAIRE-seq display	Nurminen et al., 2018
*CLMN*	Yes	ChIP-seq & FAIRE-seq display	Nurminen et al., 2018
*TMEM37*	Yes	ChIP-seq & FAIRE-seq display	Nurminen et al., 2018
*CD14*	Yes	ChIP-seq & FAIRE-seq display	Seuter et al., 2018
*COQ3*	No	ChIP-seq & FAIRE-seq display	Seuter et al., 2018
*DND1*	Yes	ChIP-seq & FAIRE-seq display	Seuter et al., 2018
*NDUFA2*	No	ChIP-seq & FAIRE-seq display	Seuter et al., 2018
*PSMB1*	No	ChIP-seq & FAIRE-seq display	Seuter et al., 2018
*SLC25A15*	No	ChIP-seq & FAIRE-seq display	Seuter et al., 2018
*SLC52A2*	No	ChIP-seq & FAIRE-seq display	Seuter et al., 2018
*TBP*	No	ChIP-seq & FAIRE-seq display	Seuter et al., 2018
*TMCO6*	Yes	ChIP-seq & FAIRE-seq display	Seuter et al., 2018
*WDR55*	Yes	ChIP-seq & FAIRE-seq display	Seuter et al., 2018
*ZNF44*	Yes	ChIP-seq & FAIRE-seq display	Seuter et al., 2018
*ACSL1*	Yes	ChIP-seq & FAIRE-seq display, RNA-seq	Nurminen et al., 2019
*CD14*	Yes	ChIP-seq & FAIRE-seq display, RNA-seq	Nurminen et al., 2019
*CDA*	No	ChIP-seq & FAIRE-seq display, RNA-seq	Nurminen et al., 2019
*FBP1*	Yes	ChIP-seq & FAIRE-seq display, RNA-seq	Nurminen et al., 2019
*G0S2*	Yes	ChIP-seq & FAIRE-seq display, RNA-seq	Nurminen et al., 2019
*GLIPR1*	Yes	ChIP-seq & FAIRE-seq display, RNA-seq	Nurminen et al., 2019
*INSR*	Yes	ChIP-seq & FAIRE-seq display, RNA-seq	Nurminen et al., 2019
*ITSN1*	Yes	ChIP-seq & FAIRE-seq display, RNA-seq	Nurminen et al., 2019
*KLHDC8B*	No	ChIP-seq & FAIRE-seq display, RNA-seq	Nurminen et al., 2019
*NFKBIA*	Yes	ChIP-seq & FAIRE-seq display, RNA-seq	Nurminen et al., 2019
*PNPLA1*	No	ChIP-seq & FAIRE-seq display, RNA-seq	Nurminen et al., 2019
*SERINC2*	Yes	ChIP-seq & FAIRE-seq display, RNA-seq	Nurminen et al., 2019
*SOAT1*	No	ChIP-seq & FAIRE-seq display, RNA-seq	Nurminen et al., 2019
*SSH1*	Yes	ChIP-seq & FAIRE-seq display, RNA-seq	Nurminen et al., 2019

*List of 34 studies that investigated vitamin D target genes in undifferentiated THP-1 cells by the indicated types of assays. The information whether a gene is a primary target (yes), secondary target (no) or not detected (-) by a RNA-seq time course is derived from a re-analysis (Neme et al., 2017) of the original dataset from Seuter et al. (2016).*

Next, all publically available transcriptome-wide data for 1,25(OH)_2_D_3_-stimulated, undifferentiated THP-1 cells were compared. A search of the Gene Expression Omnibus^2^ resulted in the microarray datasets GSE60102 (Heikkinen et al., 2011) and GSE52819 (Verway et al., 2013) as well as in the RNA-seq datasets GSE69284 (Seuter et al., 2016) and GSE119556 (Nurminen et al., 2019). In all four datasets undifferentiated THP-1 cells had been stimulated for 24 h with 1,25(OH)_2_D_3_. The comparison of the 3,372 significantly (*p* < 0.05) regulated genes in the microarray from 2011 (Heikkinen et al., 2011) and the 4,532 genes in the microarray from 2013 (Verway et al., 2013) indicated 1,227 common genes (Supplementary Figure S1). 695 of the latter matched with the 3,650 genes reported in the re-analyzed RNA-seq dataset from 2016 (Seuter et al., 2016; Neme et al., 2017) and 268 with the 951 genes identified in the RNA-seq dataset from 2018 (Nurminen et al., 2019). Furthermore, both RNA-seq datasets had 273 overlapping genes, 126 of which belonged to the 1,227 common genes found by microarrays.

Taken together, the four transcriptome-wide dataset agreed on 126 genes that were regulated within 24 h after a stimulation with 1,25(OH)_2_D_3_. Although all four experimental series had been performed in three biological repeats and only significantly (*p* < 0.05) modulated genes were considered, the rather low number of common genes indicates that the fluctuation in low expressed, moderately regulated genes was very high.

## Transcriptional Response of Vitamin D Target Genes

**FIGURE 2:**
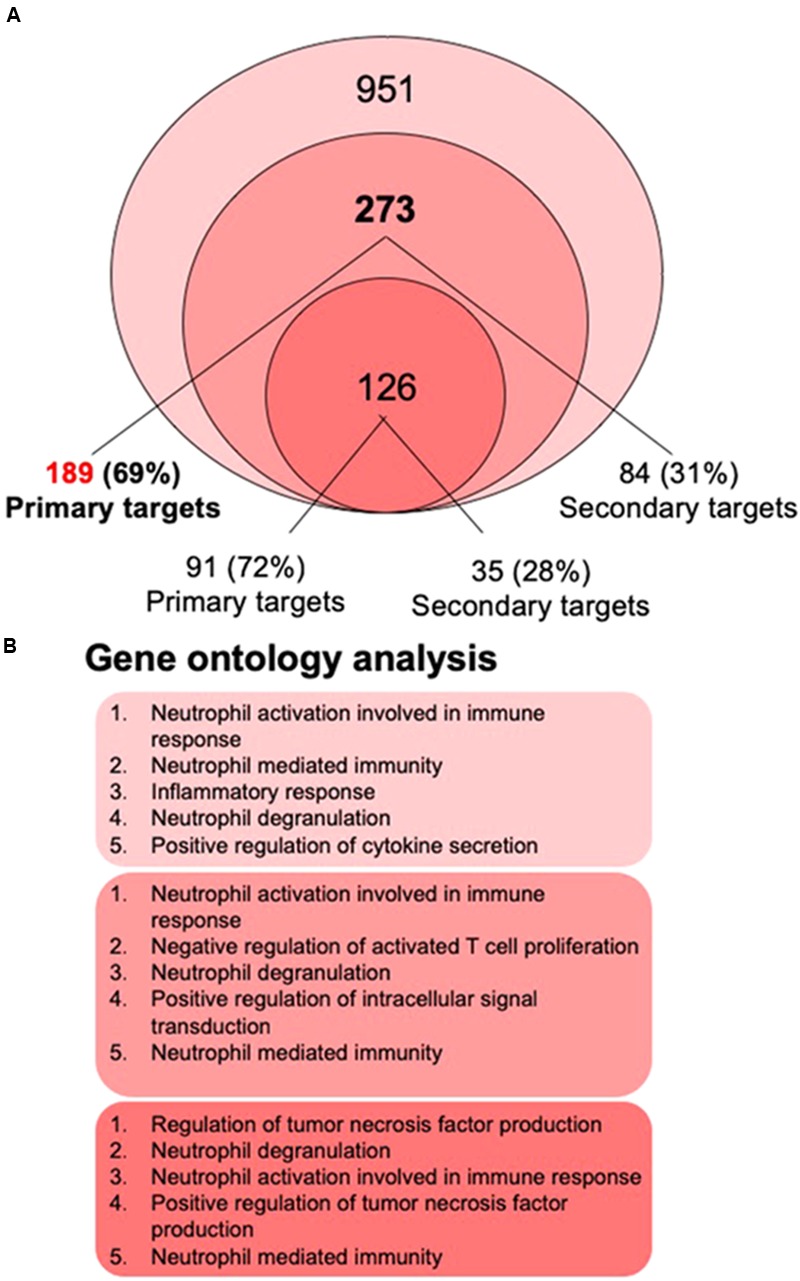
Gene ontology analysis. The most recent 1,25(OH)_2_D_3_-dependent transcriptome dataset of THP-1 cells (Nurminen et al., 2019) identified 951 genes, 273 of which overlap with the re-analyzed first RNA-seq dataset (Seuter et al., 2016; Neme et al., 2017) **(A)**. 69% of the 273 genes are primary vitamin D targets (189 genes listed in Supplementary Table S1), while from the 126 genes that were also found by microarrays 72% are primary vitamin D targets. Gene ontology analysis using the webtool *Enrichr* (Chen et al., 2013) was performed for the lists of 951, 273, and 126 members and indicated that the top five biological pathways for each of the three gene sets relate to innate immunity **(B)**.

The recent 1,25(OH)_2_D_3_-dependent transcriptome analysis (Nurminen et al., 2019) identified in THP-1 cells 951 genes as vitamin D targets, 273 of which overlapped with the re-analyzed earlier RNA-seq dataset (Seuter et al., 2016; Neme et al., 2017; Figure 2A). The latter dataset allowed assigning 189 of the 273 genes (69%) as primary vitamin D targets (Supplementary Table S1). Interestingly, 59 (75% primary) of the 273 common genes had already been characterized by single-gene approaches (Table 1). From the 126 genes that were also found by microarrays about the same proportion (72%) were primary vitamin D targets (Figure 2A).

The 189 primary vitamin D target genes (Figure 2A) were classified by the fold change (FC) of their mRNA expression after 1,25(OH)_2_D_3_ stimulation for 24 h (Supplementary Figure S2). The six genes *CD14* (encoding for a toll-like receptor co-receptor), *ORM1* (encoding for an acute phase plasma protein), *CAMP, FBP1* (encoding for a glucose metabolizing enzyme), *CYP26B1* (encoding for an enzyme metabolizing retinoids) and *TSPAN18* (encoding for a membrane protein with unclear function) showed a FC larger than 40 and form group A. The FC of further 13 genes composing group B was in the range of 10 to 40, while the large group C comprised 170 genes displaying a FC below 10. Only 5 of the 189 primary vitamin D target genes are down-regulated, while nearly half of all secondary vitamin D target genes are down-regulated (Neme et al., 2016a). Thus, in contrast to up-regulation, the mechanism of down-regulation of vitamin D target genes is mostly an indirect, multi-step process.

## Functional Profile of Vitamin D Target Genes

The biologically most important question of the transcriptome-wide analysis was, how the action of the complete set of vitamin D target genes translates into physiological functions of human monocytes. Therefore, gene ontology analysis was applied, in order to identify statistically significant overrepresentation of vitamin D target genes in biological pathways. Using the webtool *Enrichr* (Chen et al., 2013) for the gene lists with 951, 273, and 126 members (Figure 2A) provided the result that the top five biological pathways, such as “neutrophil activation,” “positive regulation of TNF production,” “inflammatory response,” “neutrophil degranulation,” “negative regulation of T cell proliferation” and “positive regulation of cytokine secretion,” related for each of the three gene sets to key functions in innate immunity (Figure 2B). This result could have been expected, since monocytes represent the major cell type of the innate immune system. Nevertheless, it is important to confirm that the key immune-related functions of monocytes are supported by vitamin D.

In summary, independent of the size of the tested target gene set, gene ontology analysis indicates that the modulation of innate immunity is the main physiological outcome of a vitamin D stimulation of human monocytes.

## Classification of Primary Vitamin D Target Genes

**FIGURE 3:**
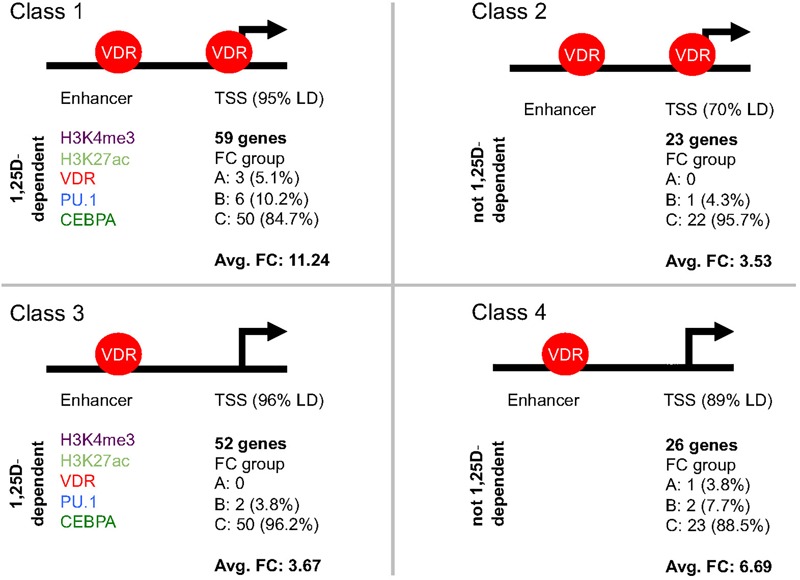
Classification of primary vitamin D target genes. The 160 primary vitamin D target genes that show H3K4me3 marks at their TSS regions can be segregated into four classes. The structure of the genes into TSS and enhancer regions is schematically depicted. Genes of classes 1 and 2 display VDR binding to their TSS regions, while for those of classes 3 and 4 no VDR binding can be detected. The 59 genes of class 1 and the 52 genes of class 3 have significant (*p* < 0.05) effects of 1,25(OH)_2_D_3_ on H3K4me3, H3K27ac, VDR, PU.1 and/or CEBPA binding strength on their enhancer region, while this is not observed for the 23 genes of class 2 and the 26 genes of class 4. In addition, for the respective classes the distribution of the genes of into the fold change (FC) groups A, B and C (Supplementary Figure S2) as well as the average FC is indicated.

Each of the 189 primary vitamin D target genes (Supplementary Table S1) was manually inspected for the epigenomic profile at its TSS and enhancer regions, such as (i) occurrence of H3K4me3 and H3K27ac marks, (ii) binding of VDR, PU.1 and CEBPA as well as (iii) the significant (*p* < 0.05) modulation of both type of datasets by 1,25(OH)_2_D_3_. The whole TAD region of each primary target gene was screened for the most prominent enhancer. For 17 genes the enhancer was found in a distance of below 1 kb from the TSS, while in contrast for 23 genes it located more than 100 kb apart (Supplementary Table S1). Importantly, 160 of the 189 genes showed H3K4me3 marks at their TSS regions and could be segregated into four classes (Supplementary Table S1 and Figure 3), which were defined as follows: VDR bound to the enhancer regions of each of these 160 genes, but only the 82 genes of classes 1 and 2 displayed VDR binding also to their TSS regions, while for the 78 genes of classes 3 and 4 no VDR was detected at their promoters. In addition to genes with enhancer regions close to their promoters (82% of which are class 1 genes), VDR could be detected at TSS regions, because it is assumed to loop from an enhancer region rather than due to direct promoter binding. Thus, probably on class 3 and 4 genes no VDR was detected, because the protein did not make long and frequent enough contacts, in order to create at the respective TSS regions a significant mark in the VDR cistrome. However, 1,25(OH)_2_D_3_-triggered increases of H3K4me3 marks at TSS regions of 56 class 3 and 4 genes provided at least indirect evidence for a looping of enhancer-bound VDR to these promoter regions.

The 59 genes of class 1 were distinguished from the 23 genes of class 2 by experiencing significant (*p* < 0.05) effects of ligand on the intensity of H3K4me3 and H3K27ac marks or the binding strength of VDR, PU.1 and/or CEBPA at their enhancer regions (Figure 3). By the same kind of 1,25(OH)_2_D_3_-sensitivity at their enhancer regions the 52 genes of class 3 differed from the 26 genes of class 4. Representative examples for the differential epigenomic profiles at enhancer and TSS regions are the regulatory scenarios of the class 1 gene *TMEM37* (encoding for a stabilizer of calcium channels), the class 2 gene *LILRB4* (encoding for a membrane receptor), the class 3 gene *TFE3* (encoding for a transcription factor) and the class 4 gene *CYP26B1* (Supplementary Figure S3). In contrast, for 29 genes no specific epigenomic pattern on their TSS and enhancer regions had been detected, i.e., they remain unclassified for their epigenomic profile (Supplementary Table S1).

The 59 genes of class 1 showed with 11.24 the highest average FC, because three of them belong to the highly up-regulated group A and six to the strongly up-regulated group B (Figure 3). Second ranking in average FC (6.69) were the 26 genes of class 4, since they comprise one group A gene and two group B genes. In contrast, the 52 class 3 genes averaged only in a FC of 3.67 (two group B genes and 50 moderately regulated group C genes) and the 23 class 2 gene even only in an average FC of 3.53 (one group B gene and 22 group C genes).

Taken together, 160 of the 189 primary vitamin D target genes could be segregated into four classes by the epigenomic profile of their enhancer and TSS regions. The key distinguishing protein of the respective gene regulatory scenarios was VDR bound to TSS and enhancer regions and being supported at enhancers by the pioneer transcription factors PU.1 and CEBPA.

## Relation of Gene Regulatory Scenarios to Biological Function

**FIGURE 4:**
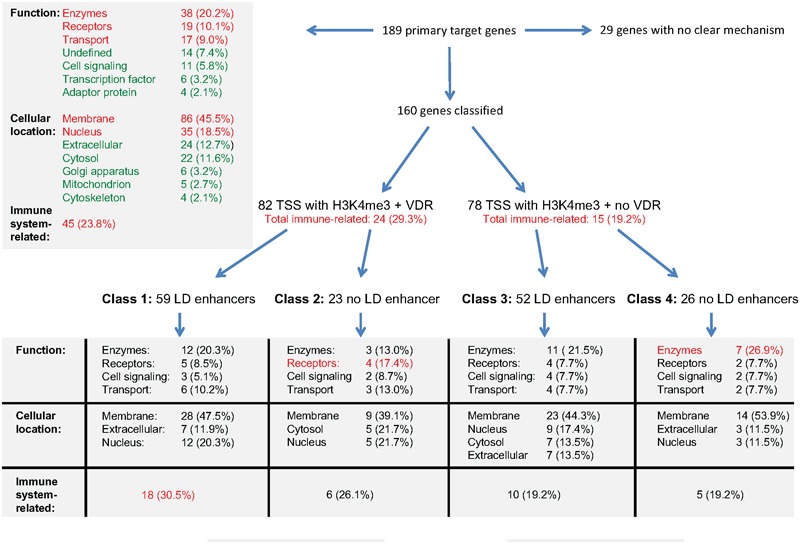
Integration of gene regulatory scenarios with biological function. The main relations between the epigenomic profiles of primary vitamin D target genes and the function, cellular location and relation to immunity of their encoded proteins is outlined. More details are provided in the text.

The integration of the transcriptome- and epigenome-wide datasets available for vitamin D-triggered human monocytes can be summarized as outlined in Figure 4. On the transcriptome-level, a stimulation of THP-1 cells with 1,25(OH)_2_D_3_ has significant (*p* < 0.05) direct effects on the expression of 189 genes, 184 of which are up-regulated (Supplementary Table S1). The proteins encoded by these genes mainly act as enzymes (20%), receptors (10%) and transporters (9%) and their most prominent locations are membranes (46%) and the nucleus (19%). Nearly a fourth (23.8%) of these primary vitamin D target genes are directly involved in the function of the immune system. On the epigenome level, 160 of the 189 genes can be classified into four gene regulatory scenarios, while 29 genes (15%) remain unclassified. The 82 genes with VDR binding at their TSS region show with 29.3% a higher rate of immune system related function than the 78 genes without detectable VDR (19.2%). Accordingly, the main characteristic of the 59 class 1 genes is the relation of their function to the immune system. In contrast, in absence of significant effects of 1,25(OH)_2_D_3_ on enhancer regions, as it applies for classes 2 and 4, preferentially genes with metabolic functions are found.

In summary, immune system-related genes of class 1 are supported by a gene regulatory scenario that allows a prominent up-regulation by vitamin D, while genes related to cellular metabolism show less vitamin D sensitive epigenomic profiles.

## Future Perspective: Translation to Primary Cells

In the vitamin D intervention study VitDbol (NCT02063334, ClinicalTrials.gov) healthy human adults were treated once with high dose of vitamin D_3_ (2,000 μg) and blood samples were taken before and 24 h later (Vukic et al., 2015). Peripheral blood mononuclear cells (PBMCs, a mixture of the vitamin D-responsive cell types monocytes, T and B lymphocytes) were isolated in less than an hour after drawing blood, i.e., the epigenome and transcriptome status of the cells was as close as possible to the *in vivo* situation (Carlberg, 2016). Excluding any further *in vitro* culture, RNA and chromatin were prepared, in order to test changes in gene expression by qPCR (Vukic et al., 2015) and RNA-seq (Neme et al., 2019) as well as alterations in chromatin accessibility at selected genomic regions (Seuter et al., 2017b) and genome-wide (Carlberg et al., 2018). Based on five tested individuals the expression of 702 genes changed significantly (*p* < 0.05) in response to vitamin D_3_ (Neme et al., 2019), 26% of which were also observed in THP-1 cells as vitamin D target genes (Neme et al., 2017). Although the latter genes were primarily secondary targets, it indicates that the response of PBMCs *in vivo* to a vitamin D_3_ bolus resembles that of THP-1 cells *in vitro* to a 1,25(OH)_2_D_3_ bolus. Similarly, the vitamin D_3_ bolus significantly (*p* < 0.05) changes *in vivo* the chromatin accessibility at 853 genomic regions (Carlberg et al., 2018), 87% of which were also 1,25(OH)_2_D_3_ responsive in THP-1 cells (Seuter et al., 2016).

Taken together, there is reasonable overlap in the transcriptome- and epigenome-wide vitamin D response of PBMCs *in vivo* and THP-1 cells *in vitro*, i.e., principles of vitamin D signaling described in this review for THP-1 cells may also be extrapolated to primary cells, such as PBMCs. Primary monocytes would be most appropriate cellular target, for which, however, no vitamin D-triggered epigenome-wide data exists.

## Conclusion

To date, most epigenome- and transcriptome-wide data are available for the actions of vitamin D in cells of the hematopoietic system (Carlberg, 2014). The integration of these large datasets led to the chromatin model of vitamin D signaling (Carlberg, 2017), which was extended in this summary of recent data by the introduction of four different classes of primary vitamin D target genes. The respective gene regulatory scenarios distinguish prominent vitamin D response of genes related to immune function and moderate response of genes involved in cellular metabolism.

## Author Contributions

VN and CC designed the project. VN, SS, and CC analyzed the data. CC wrote the manuscript.

## Conflict of Interest Statement

The authors declare that the research was conducted in the absence of any commercial or financial relationships that could be construed as a potential conflict of interest.

## Footnotes

1 www.proteinatlas.org/ENSG00000111424-VDR/tissue

2 www.ncbi.nlm.nih.gov/geo

## Abbreviations

1,25(OH)_2_D_3_: 1α,25-dihydroxyvitamin D_3_
CAMP: cathelicidin antimicrobial peptide
CD14: CD14 molecule
CEBPA: CCAAT/enhancer binding protein alpha
ChIP-seq: chromatin immunoprecipitation sequencing
CTCF: CCCTC-binding factor
CYP26B1: cytochrome P450 family 26 subfamily B member 1
DR3: direct repeat spaced by 3 nucleotides
FAIRE-seq: formaldehyde-assisted isolation of regulatory elements sequencing
FBP1: fructose-bisphosphatase 1
FC: fold change
IGV: Integrative Genomics Viewer
LD: ligand-dependent
LILRB4: leukocyte immunoglobulin like receptor B4
ORM1: orosomucoid 1
PBMC: peripheral blood mononuclear cells
PU.1: purine-rich box 1
RNA-seq: RNA sequencing
RXR: retinoid X receptor
Spi-1 proto-oncogene: (official gene symbol: *SPI1*)
TAD: topologically associated domain
TFE3: transcription factor binding to IGHM enhancer 3
TMEM37: transmembrane protein 37
TSPAN18: tetraspanin 18
TSS: transcription start site
VDR: vitamin D receptor

## References

[B1] Ali T. Renkawitz R. Bartkuhn M. (2016). Insulators and domains of gene expression. *Curr. Opin. Genet. Dev.* 37 17–26. 10.1016/j.gde.2015.11.009 26802288

[B2] Beisel C. Paro R. (2011). Silencing chromatin: comparing modes and mechanisms. *Nat. Rev. Genet.* 12 123–135. 10.1038/nrg2932 21221116

[B3] Bendik I. Friedel A. Roos F. F. Weber P. Eggersdorfer M. (2014). Vitamin D: a critical and essential micronutrient for human health. *Front. Physiol.* 5:248 10.3389/fphys.2014.00248 PMC4092358 25071593

[B4] Bouillon R. Suda T. (2014). Vitamin D: calcium and bone homeostasis during evolution. *Bonekey Rep.* 3:480. 10.1038/bonekey.2013.214 24466411 PMC3899559

[B5] Carlberg C. (2014). Genome-wide (over)view on the actions of vitamin D. *Front. Physiol.* 5:167. 10.3389/fphys.2014.00167 24808867 PMC4010781

[B6] Carlberg C. (2016). Molecular approaches for optimizing vitamin D supplementation. *Vitam. Horm.* 100 255–271. 10.1016/bs.vh.2015.10.001 26827955

[B7] Carlberg C. (2017). Molecular endocrinology of vitamin D on the epigenome level. *Mol. Cell. Endocrinol.* 453 14–21. 10.1016/j.mce.2017.03.016 28315703

[B8] Carlberg C. (2018). Vitamin D genomics: from in vitro to in vivo. *Front. Endocrinol.* 9:250 10.3389/fendo.2018.00250 PMC5974042 29875733

[B9] Carlberg C. Bendik I. Wyss A. Meier E. Sturzenbecker L. J. Grippo J. F. (1993). Two nuclear signalling pathways for vitamin D. *Nature* 361 657–660. 10.1038/361657a0 8382345

[B10] Carlberg C. Campbell M. J. (2013). Vitamin D receptor signaling mechanisms: integrated actions of a well-defined transcription factor. *Steroids* 78 127–136. 10.1016/j.steroids.2012.10.019 23178257 PMC4668715

[B11] Carlberg C. Molnár F. (2016a). *The Epigenome. Mechanisms of Gene Regulation* 2nd Edn. Berlin: Springer 159–172.

[B12] Carlberg C. Molnár F. (2016b). *The Impact of Chromatin. Mechanisms of Gene Regulation* 2nd Edn Berlin: Springer 17–34.

[B13] Carlberg C. Seuter S. Nurmi T. Tuomainen T. P. Virtanen J. K. Neme A. (2018). In vivo response of the human epigenome to vitamin D: a proof-of-principle study. *J. Steroid. Biochem. Mol. Biol.* 180 142–148. 10.1016/j.jsbmb.2018.01.002 29317287

[B14] Chen E. Y. Tan C. M. Kou Y. Duan Q. Wang Z. Meirelles G. V. (2013). Enrichr: interactive and collaborative HTML5 gene list enrichment analysis tool. *BMC Bioinformatics* 14:128. 10.1186/1471-2105-14-128 23586463 PMC3637064

[B15] Dawson M. A. Kouzarides T. (2012). Cancer epigenetics: from mechanism to therapy. *Cell* 150 12–27. 10.1016/j.cell.2012.06.013 22770212

[B16] Ding N. Yu R. T. Subramaniam N. Sherman M. H. Wilson C. Rao R. (2013). A vitamin D receptor/SMAD genomic circuit gates hepatic fibrotic response. *Cell* 153 601–613. 10.1016/j.cell.2013.03.028 23622244 PMC3673534

[B17] Dixon J. R. Selvaraj S. Yue F. Kim A. Li Y. Shen Y. (2012). Topological domains in mammalian genomes identified by analysis of chromatin interactions. *Nature* 485 376–380. 10.1038/nature11082 22495300 PMC3356448

[B18] Gombart A. F. Borregaard N. Koeffler H. P. (2005). Human cathelicidin antimicrobial peptide (CAMP) gene is a direct target of the vitamin D receptor and is strongly up-regulated in myeloid cells by 1,25-dihydroxyvitamin D3. *FASEB J.* 19 1067–1077. 10.1096/fj.04-3284com 15985530

[B19] Grad R. (2004). Cod and the consumptive: a brief history of cod-liver oil in the treatment of pulmonary tuberculosis. *Pharm. Hist.* 46 106–120. 15712453

[B20] Hart P. H. Gorman S. Finlay-Jones J. J. (2011). Modulation of the immune system by UV radiation: more than just the effects of vitamin D? *Nat. Rev. Immunol.* 11 584–596. 10.1038/nri3045 21852793

[B21] Haussler M. R. Whitfield G. K. Kaneko I. Haussler C. A. Hsieh D. Hsieh J.-C. (2013). Molecular mechanisms of vitamin D action. *Calcif. Tissue Int.* 92 77–98. 10.1007/s00223-012-9619-0 22782502

[B22] Heikkinen S. Väisänen S. Pehkonen P. Seuter S. Benes V. Carlberg C. (2011). Nuclear hormone 1α,25-dihydroxyvitamin D3 elicits a genome-wide shift in the locations of VDR chromatin occupancy. *Nucleic Acids Res.* 39 9181–9193. 10.1093/nar/gkr654 21846776 PMC3241659

[B23] Herdick M. Carlberg C. (2000). Agonist-triggered modulation of the activated and silent state of the vitamin D3 receptor by interaction with co-repressors and co-activators. *J. Mol. Biol.* 304 793–801. 10.1006/jmbi.2000.4267 11124027

[B24] Hewison M. (2012). An update on vitamin D and human immunity. *Clin. Endocrinol.* 76 315–325. 10.1111/j.1365-2265.2011.04261.x 21995874

[B25] Holick M. F. (1981). The cutaneous photosynthesis of previtamin D3: a unique photoendocrine system. *J. Invest Ermatol.* 77 51–58. 10.1111/1523-1747.ep12479237 6265564

[B26] Holick M. F. (2011). *Photobiology of Vitamin D.* Boston, MA: Boston University 13–22. 10.1016/B978-0-12-381978-9.10002-2

[B27] Hosoda H. Tamura H. Nagaoka I. (2015). Evaluation of the lipopolysaccharide-induced transcription of the human TREM-1 gene in vitamin D3-matured THP-1 macrophage-like cells. *Int. J. Mol. Med.* 36 1300–1310. 10.3892/ijmm.2015.2349 26397033

[B28] Liu P. T. Stenger S. Li H. Wenzel L. Tan B. H. Krutzik S. R. (2006). Toll-like receptor triggering of a vitamin D-mediated human antimicrobial response. *Science* 311 1770–1773. 10.1126/science.1123933 16497887

[B29] Liu P. T. Stenger S. Tang D. H. Modlin R. L. (2007). Cutting edge: vitamin D-mediated human antimicrobial activity against *Mycobacterium tuberculosis* Is dependent on the induction of cathelicidin. *J. Immunol.* 179 2060–2063. 10.4049/jimmunol.179.4.2060 17675463

[B30] Matsunawa M. Amano Y. Endo K. Uno S. Sakaki T. Yamada S. (2009). The aryl hydrocarbon receptor activator benzo[a]pyrene enhances vitamin D3 catabolism in macrophages. *Toxicol. Sci.* 109 50–58. 10.1093/toxsci/kfp044 19244278

[B31] Meyer M. B. Goetsch P. D. Pike J. W. (2012). VDR/RXR and TCF4/beta-catenin cistromes in colonic cells of colorectal tumor origin: impact on c-FOS and c-MYC gene expression. *Mol. Endocrinol.* 26 37–51. 10.1210/me.2011-1109 22108803 PMC3248320

[B32] Moeenrezakhanlou A. Shephard L. Lam L. Reiner N. E. (2008). Myeloid cell differentiation in response to calcitriol for expression CD11b and CD14 is regulated by myeloid zinc finger-1 protein downstream of phosphatidylinositol 3-kinase. *J. Leukoc. Biol.* 84 519–528. 10.1189/jlb.1207833 18495781

[B33] Molnár F. Peräkylä M. Carlberg C. (2006). Vitamin D receptor agonists specifically modulate the volume of the ligand-binding pocket. *J. Biol. Chem.* 281 10516–10526. 10.1074/jbc.M513609200 16478719

[B34] Neme A. Nurminen V. Seuter S. Carlberg C. (2016a). The vitamin D-dependent transcriptome of human monocytes. *J. Steroid Biochem. Mol. Biol.* 164 180–187. 10.1016/j.jsbmb.2015.10.018 26523676

[B35] Neme A. Seuter S. Carlberg C. (2016b). Vitamin D-dependent chromatin association of CTCF in human monocytes. *Biochim. Biophys. Acta* 1859 1380–1388. 10.1016/j.bbagrm.2016.08.008 27569350

[B36] Neme A. Seuter S. Carlberg C. (2017). Selective regulation of biological processes by vitamin D based on the spatio-temporal cistrome of its receptor. *Biochim. Biophys. Acta* 1860 952–961. 10.1016/j.bbagrm.2017.07.002 28712921

[B37] Neme A. Seuter S. Malinen M. Nurmi T. Tuomainen T. P. Virtanen J. K. (2019). *In vivo* transcriptome changes of human white blood cells in response to vitamin D. *J. Steroid Biochem. Mol. Biol.* 10.1016/j.jsbmb.2018.11.019 [Epub ahead of print]. 30537545

[B38] Nurminen V. Neme A. Ryynanen J. Heikkinen S. Seuter S. Carlberg C. (2015). The transcriptional regulator BCL6 participates in the secondary gene regulatory response to vitamin D. *Biochim. Biophys. Acta* 1849 300–308. 10.1016/j.bbagrm.2014.12.001 25482012

[B39] Nurminen V. Neme A. Seuter S. Carlberg C. (2018). The impact of the vitamin D-modulated epigenome on VDR target gene regulation. *Biochim. Biophys. Acta* 1861 697–705. 10.1016/j.bbagrm.2018.05.006 30018005

[B40] Nurminen V. Neme A. Seuter S. Carlberg C. (2019). Modulation of vitamin D signaling by the pioneer factor CEBPA. *Biochim. Biophys. Acta* 1862 96–106. 10.1016/j.bbagrm.2018.12.004 30550771

[B41] Pereira F. Barbachano A. Silva J. Bonilla F. Campbell M. J. Munoz A. (2011). DM6B/JMJD3 histone demethylase is induced by vitamin D and modulates its effects in colon cancer cells. *Hum. Mol. Genet.* 20 4655–4665. 10.1093/hmg/ddr399 21890490

[B42] Polly P. Herdick M. Moehren U. Baniahmad A. Heinzel T. Carlberg C. (2000). VDR-Alien: a novel, DNA-selective vitamin D3 receptor-corepressor partnership. *FASEB J.* 14 1455–1463. 10.1096/fasebj.14.10.1455 10877839

[B43] Ramagopalan S. V. Heger A. Berlanga A. J. Maugeri N. J. Lincoln M. R. Burrell A. (2010). A ChIP-seq defined genome-wide map of vitamin D receptor binding: associations with disease and evolution. *Genome Res.* 20 1352–1360. 10.1101/gr.107920.110 20736230 PMC2945184

[B44] Reeves R. K. Takahashi H. Hatta Y. Iriyama N. Hasegawa Y. Uchida H. (2014). Induced differentiation of human myeloid leukemia cells into M2 macrophages by combined treatment with retinoic acid and 1α,25-dihydroxyvitamin D3. *PLoS One* 9:e113722. 10.1371/journal.pone.0113722 25409436 PMC4237509

[B45] Rook G. A. Steele J. Fraher L. Barker S. Karmali R. O’Riordan J. (1986). Vitamin D3, gamma interferon, and control of proliferation of Mycobacterium tuberculosis by human monocytes. *Immunology* 57 159–163. 3002968 PMC1453883

[B46] Seuter S. Heikkinen S. Carlberg C. (2013a). Chromatin acetylation at transcription start sites and vitamin D receptor binding regions relates to effects of 1α,25-dihydroxyvitamin D3 and histone deacetylase inhibitors on gene expression. *Nucleic Acids Res.* 41 110–124. 10.1093/nar/gks959 23093607 PMC3592476

[B47] Seuter S. Neme A. Carlberg C. (2014a). Characterization of genomic vitamin D receptor binding sites through chromatin looping and opening. *PLoS One* 9:e96184. 10.1371/journal.pone.0096184 24763502 PMC3999108

[B48] Seuter S. Neme A. Carlberg C. (2016). Epigenome-wide effects of vitamin D and their impact on the transcriptome of human monocytes involve CTCF. *Nucleic Acids Res.* 44 4090–4104. 10.1093/nar/gkv1519 26715761 PMC4872072

[B49] Seuter S. Neme A. Carlberg C. (2017a). Epigenomic PU.1-VDR crosstalk modulates vitamin D signaling. *Biochim. Biophys. Acta* 1860 405–415. 10.1016/j.bbagrm.2017.02.005 28232093

[B50] Seuter S. Neme A. Carlberg C. (2018). ETS transcription factor family member GABPA contributes to vitamin D receptor target gene regulation. *J. Steroid Biochem. Mol. Biol.* 177 46–52. 10.1016/j.jsbmb.2017.08.006 28870774

[B51] Seuter S. Pehkonen P. Heikkinen S. Carlberg C. (2013b). Dynamics of 1α,25-dihydroxyvitamin D-dependent chromatin accessibility of early vitamin D receptor target genes. *Biochim. Biophys. Acta* 1829 1266–1275. 10.1016/j.bbagrm.2013.10.003 24185200

[B52] Seuter S. Pehkonen P. Heikkinen S. Carlberg C. (2013c). The gene for the transcription factor BHLHE40/DEC1/stra13 is a dynamically regulated primary target of the vitamin D receptor. *J. Steroid Biochem. Mol. Biol.* 136 62–67. 10.1016/j.jsbmb.2012.11.011 23220548

[B53] Seuter S. Ryynänen J. Carlberg C. (2014b). The ASAP2 gene is a primary target of 1,25-dihydroxyvitamin D in human monocytes and macrophages. *J. Steroid Biochem. Mol. Biol.* 144 12–18. 10.1016/j.jsbmb.2013.08.014 23999061

[B54] Seuter S. Virtanen J. K. Nurmi T. Pihlajamäki J. Mursu J. Voutilainen S. (2017b). Molecular evaluation of vitamin D responsiveness of healthy young adults. *J. Steroid Biochem. Mol. Biol.* 174 314–321. 10.1016/j.jsbmb.2016.06.003 27282116

[B55] The Encode-Project-Consortium Bernstein B. E. Birney E. Dunham I. Green E. D. Gunter C. (2012). An integrated encyclopedia of DNA elements in the human genome. *Nature* 489 57–74. 10.1038/nature11247 22955616 PMC3439153

[B56] Tsuchiya S. Yamabe M. Yamaguchi Y. Kobayashi Y. Konno T. Tada K. (1980). Establishment and characterization of a human acute monocytic leukemia cell line (THP-1). *Int. J. Cancer* 26 171–176. 10.1002/ijc.2910260208 6970727

[B57] Tuoresmäki P. Väisänen S. Neme A. Heikkinen S. Carlberg C. (2014). Patterns of genome-wide VDR locations. *PLoS One* 9:e96105. 10.1371/journal.pone.0096105 24787735 PMC4005760

[B58] Verma R. Jung J. H. Kim J. Y. (2014). 1,25-Dihydroxyvitamin D3 up-regulates TLR10 while down-regulating TLR2, 4, and 5 in human monocyte THP-1. *J. Steroid Biochem. Mol. Biol.* 141 1–6. 10.1016/j.jsbmb.2013.12.012 24373795

[B59] Verway M. Bouttier M. Wang T. T. Carrier M. Calderon M. An B. S. (2013). Vitamin D induces interleukin-1beta expression: paracrine macrophage epithelial signaling controls *M. tuberculosis* infection. *PLoS Pathog.* 9:e1003407. 10.1371/journal.ppat.1003407 23762029 PMC3675149

[B60] Vukic M. Neme A. Seuter S. Saksa N. de Mello V. D. Nurmi T. (2015). Relevance of vitamin D receptor target genes for monitoring the vitamin D responsiveness of primary human cells. *PLoS One* 10:e0124339. 10.1371/journal.pone.0124339 25875760 PMC4395145

[B61] Wei Z. Yoshihara E. He N. Hah N. Fan W. Pinto A. F. M. (2018). Vitamin D switches BAF complexes to protect beta cells. *Cell* 173 1135–1149.e15. 10.1016/j.cell.2018.04.013 29754817 PMC5987229

[B62] Wilfinger J. Seuter S. Tuomainen T.-P. Virtanen J. K. Voutilainen S. Nurmi T. (2014). Primary vitamin D receptor target genes as biomarkers for the vitamin D3 status in the hematopoietic system. *J. Nutr. Biochem.* 25 875–884. 10.1016/j.jnutbio.2014.04.002 24854954

[B63] Wu S. Ren S. Nguyen L. Adams J. S. Hewison M. (2007). Splice variants of the CYP27b1 gene and the regulation of 1,25-dihydroxyvitamin D3 production. *Endocrinology* 148 3410–3418. 10.1210/en.2006-1388 17395703

