# Supplementary material for: Primary Vitamin D Target Genes of Human Monocytes
Source: Front Physiol. 2019 Mar 5;10:194. doi: 10.3389/fphys.2019.00194 (PMC6411690; doi:10.3389/fphys.2019.00194)
Supplement: Supplementary file 1 [file Data_Sheet_1.PDF]

SUPPLEMENTARY TABLE

Table S1: Classification of primary vitamin D target genes. Characterization of 189 primary vitamin D target genes (Nurminen et al., 2018b) by the epigenomic properties of their TSS and enhancer regions, such as ChIP-seq peaks for H3K4me3, H3K27ac, VDR, PU.1 and CEBPA and their ligand-dependency (LD), distance of the enhancer from the TSS, their transcriptomic properties, such as fold change (FC) after 24 stimulation with 1,25(OH)$_2$D$_3$, and their functional attributes, such as gene function, relation to immune system and cellular location (all based on GeneCards (www.genecards.org)). Color shading marks the members of class 1 (blue), class 2 (green), class 3 (purple) and class 4 (brown); 29 genes could not be classified and stayed non-shaded.

SUPPLEMENTARY FIGURES

Figure S1: Vitamin D target gene overlap in transcriptome-wide datasets. Venn diagrams compare significantly ($p < 0.05$) 1,25(OH)$_2$D$_3$-sensitive genes of the microarrays GSE60102 (Heikkinen et al., 2011) and GSE52819 (Verway et al., 2013) (**top**). The 1,227 common genes of the microarrays were related to the RNA-seq datasets GSE119556 (Nurminen et al., 2018b) (**center, left**) and GSE69284 (Seuter et al., 2016) (**center, right**) as well to the 273 common genes of both RNA-seq datasets (**bottom**).

Figure S2: Inducibility of primary vitamin D target genes. The 189 primary vitamin D target genes revealed by RNA-seq (Nurminen et al., 2018b) are sorted by descending fold change (FC) in mRNA expression. They are classified into three groups: A (FC > 40), B (FC 10-40) and C (FC < 10).

Figure S3: Genomic view of epigenomic profiles. The Integrative Genomics Viewer (IGV) browser (Thorvaldsdottir et al., 2013) was used to display the epigenomic profiles at enhancer and TSS regions of the genes *TMEM37* (class 1), *LILRB4* (class 2), *TFE3* (class 3) and *CYP26B1* (class 4). The peak tracks display merged data from three biological repeats of ChIP-seq experiments with antibodies against VDR (red), H3K4me3 (purple), H3K27ac (light brown), PU.1 (blue) and CEBPA (green) obtained in THP-1 cells that were treated for 24 h with vehicle (EtOH) or 1,25(OH)$_2$D$_3$ (1,25D). FAIRE-seq data (light blue) serve as a reference. The gene structures are shown in blue and the fold change (FC) of gene expression is indicated below.

Fig. S1

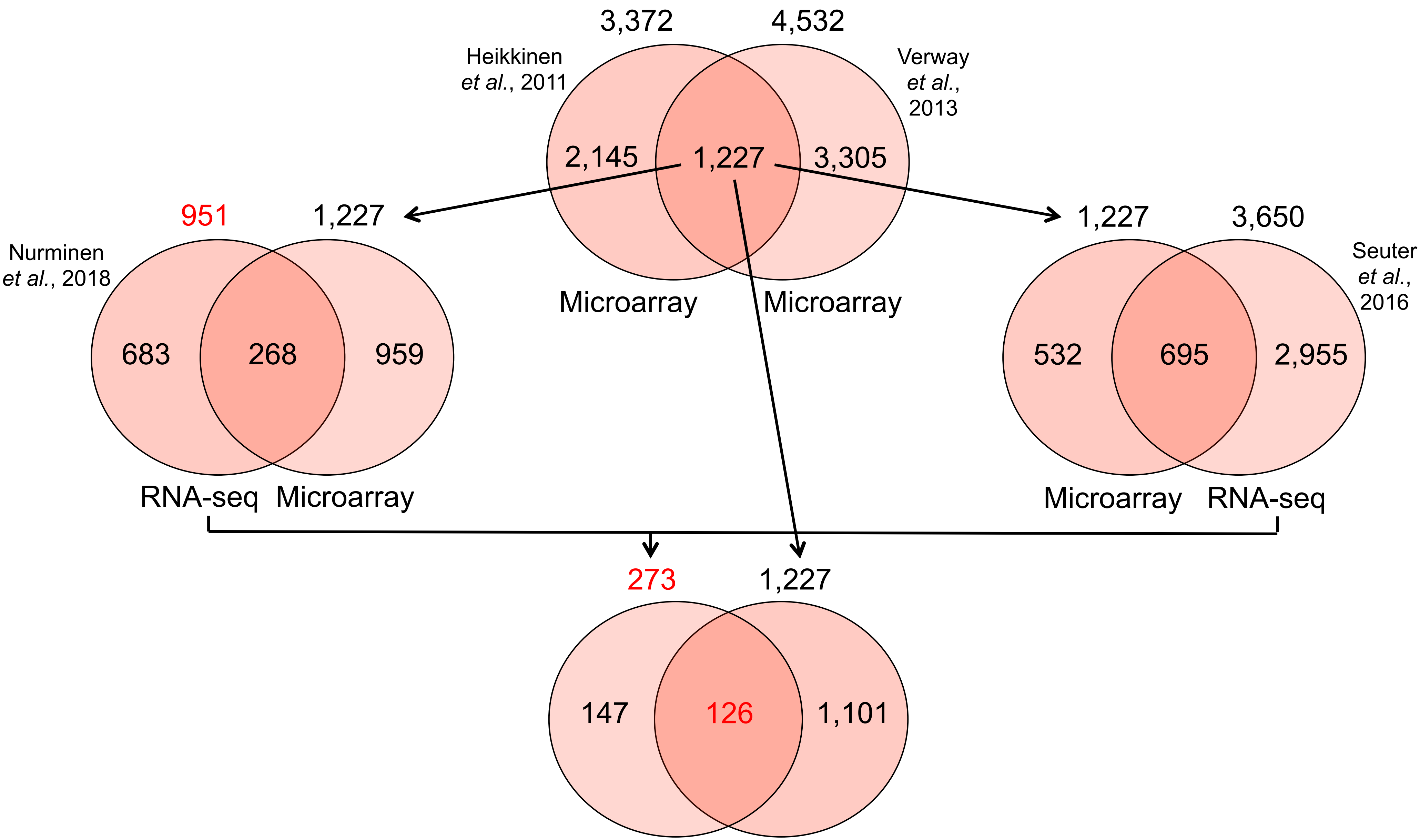

3,372
4,532
Heikkinen
et al., 2011
Verway
et al.,
2013
2,145
1,227
3,305
Microarray
Microarray
951
1,227
Nurminen
et al., 2018
683
268
959
RNA-seq
Microarray
1,227
3,650
Seuter
et al.,
2016
532
695
2,955
Microarray
RNA-seq
273
1,227
147
126
1,101

Fig. S2

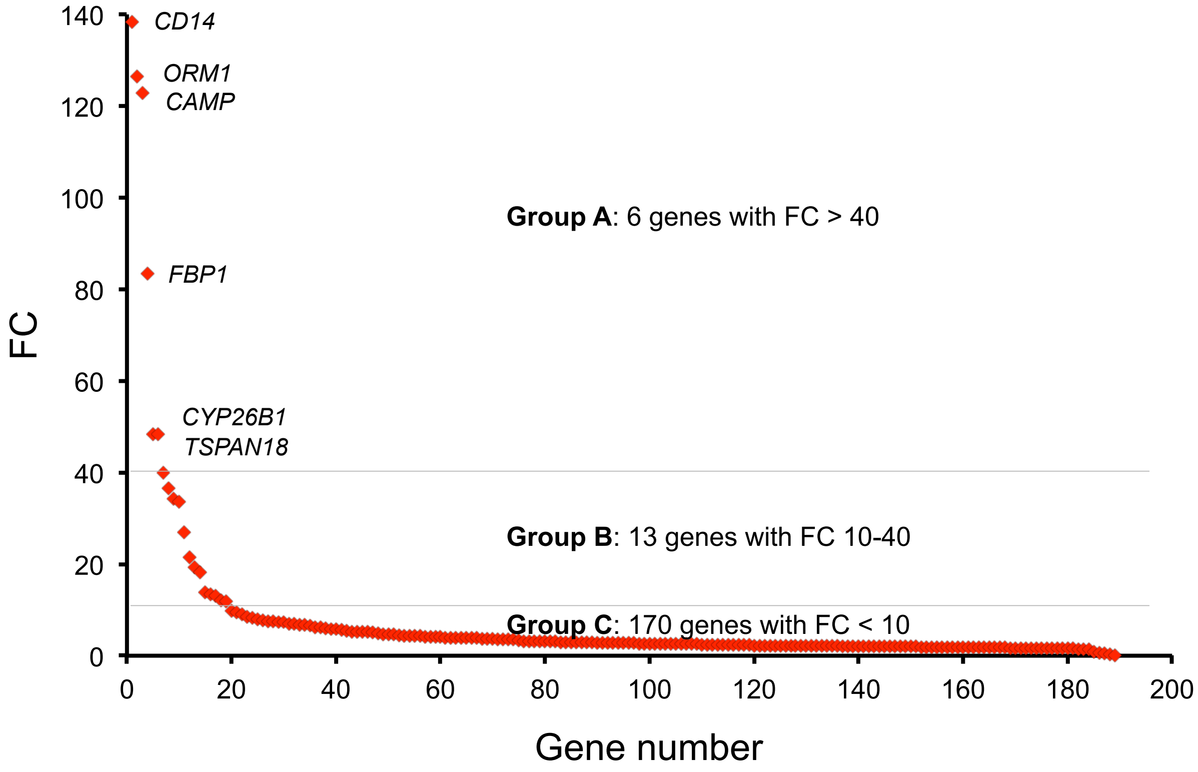

140
120
100
80
60
40
20
0
FC
CD14
ORM1
CAMP
FBP1
CYP26B1
TSPAN18
Group A: 6 genes with FC > 40
Group B: 13 genes with FC 10-40
Group C: 170 genes with FC < 10
0
20
40
60
80
100
120
140
160
180
200
Gene number

Fig. S3

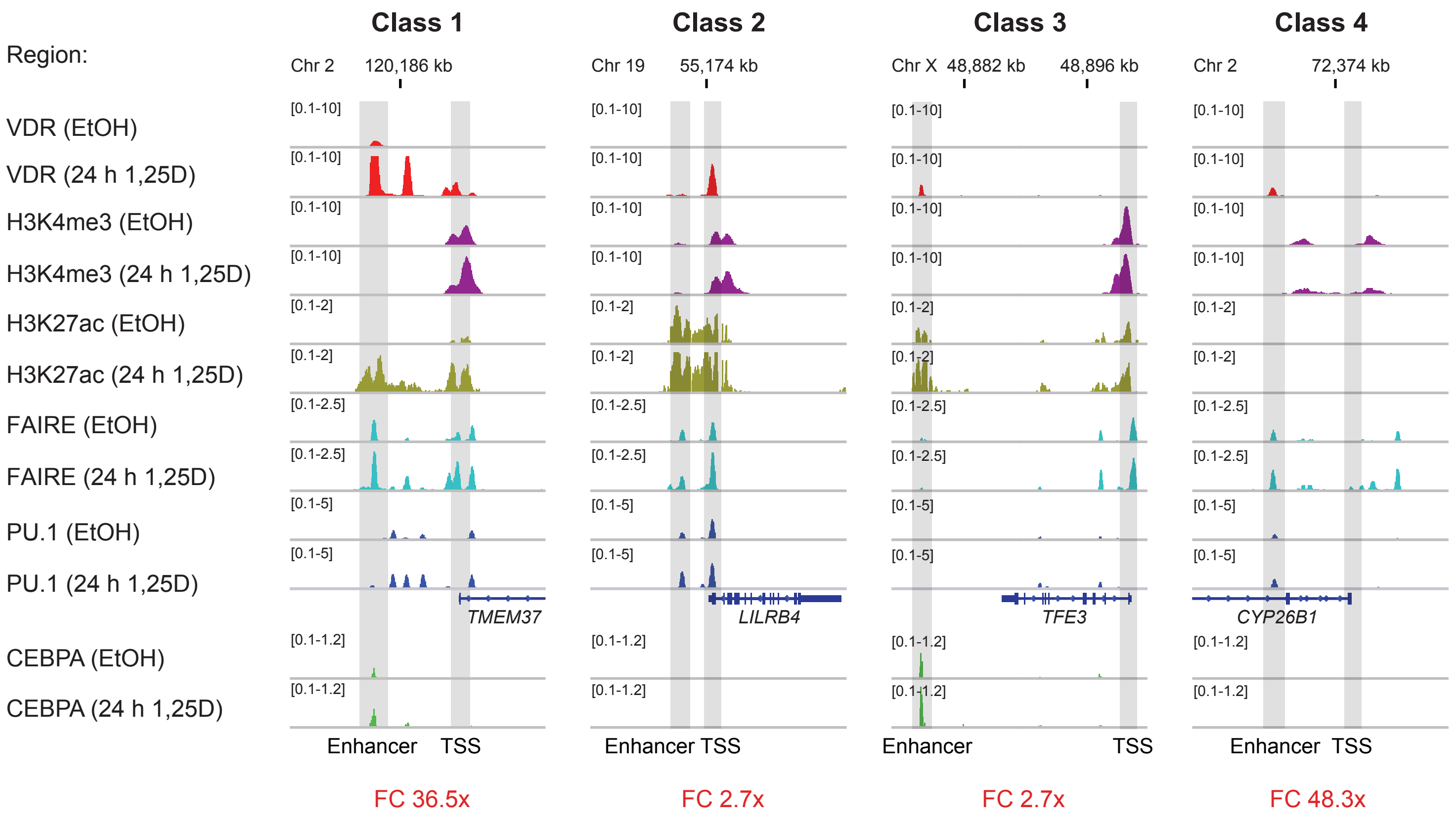
Class 1
Class 2
Class 3
Class 4
Region:
Chr 2 120,186 kb
Chr 19 55,174 kb
Chr X 48,882 kb 48,896 kb
Chr 2 72,374 kb
VDR (EtOH)
VDR (24 h 1,25D)
H3K4me3 (EtOH)
H3K4me3 (24 h 1,25D)
H3K27ac (EtOH)
H3K27ac (24 h 1,25D)
FAIRE (EtOH)
FAIRE (24 h 1,25D)
PU.1 (EtOH)
PU.1 (24 h 1,25D)
CEBPA (EtOH)
CEBPA (24 h 1,25D)
[0.1-10]
[0.1-2]
[0.1-2.5]
[0.1-5]
[0.1-1.2]
TMEM37
LILRB4
TFE3
CYP26B1
Enhancer TSS
Enhancer TSS
Enhancer TSS
Enhancer TSS
FC 36.5x
FC 2.7x
FC 2.7x
FC 48.3x
